# The Social Perception of Heroes and Murderers: Effects of Gender-Inclusive Language in Media Reports

**DOI:** 10.3389/fpsyg.2016.00369

**Published:** 2016-03-22

**Authors:** Karolina Hansen, Cindy Littwitz, Sabine Sczesny

**Affiliations:** ^1^Faculty of Psychology, University of WarsawWarsaw, Poland; ^2^Friedrich-Schiller-University JenaJena, Germany; ^3^University of BernBern, Switzerland

**Keywords:** heroism, crime, murder, gender, social roles, gender-fair language, cognitive availability, newspaper reports

## Abstract

The way media depict women and men can reinforce or diminish gender stereotyping. Which part does language play in this context? Are roles perceived as more gender-balanced when feminine role nouns are used in addition to masculine ones? Research on gender-inclusive language shows that the use of feminine-masculine word pairs tends to increase the visibility of women in various social roles. For example, when speakers of German were asked to name their favorite “heroine or hero in a novel,” they listed more female characters than when asked to name their favorite “hero in a novel.” The research reported in this article examines how the use of gender-inclusive language in news reports affects readers’ own usage of such forms as well as their mental representation of women and men in the respective roles. In the main experiment, German participants (*N* = 256) read short reports about heroes or murderers which contained either masculine generics or gender-inclusive forms (feminine-masculine word pairs). Gender-inclusive forms enhanced participants’ own usage of gender-inclusive language and this resulted in more gender-balanced mental representations of these roles. Reading about “heroines and heroes” made participants assume a higher percentage of women among persons performing heroic acts than reading about “heroes” only, but there was no such effect for murderers. A post-test suggested that this might be due to a higher accessibility of female exemplars in the category heroes than in the category murderers. Importantly, the influence of gender-inclusive language on the perceived percentage of women in a role was mediated by speakers’ own usage of inclusive forms. This suggests that people who encounter gender-inclusive forms and are given an opportunity to use them, use them more themselves and in turn have more gender-balanced mental representations of social roles.

## Introduction

When we open a newspaper, we often encounter headlines such as “Hometown driver now a local hero” or “A lot of heroes around here.” We may also come across a headline that reads “While at war, female soldiers fight to belong” (New York Times, 25 May 2015). What images of men and women do such newspaper articles create? Does the language they contain influence these images? In our research, we studied the effects of using either only masculine or both masculine and feminine role nouns in newspaper articles. A large body of research documents that women are less visible in the media in general: only 13% of all news stories are about women (Macharia et al., 2010). Furthermore, the media often depict women and men in a stereotyped manner, with 46% of news stories reinforcing gender stereotypes, and only 6% challenging such stereotypes (Macharia et al., 2010). Gender stereotypes that prevail in a society are reflected in the media, but the media also influence how women and men are perceived in the respective society. Also, the way research findings are reported in the popular press may affect readers’ beliefs and attitudes and may reinforce stereotyping. For example, a series of studies showed that readers of an article that stressed biological explanations of gender differences endorsed gender stereotypes more strongly than readers of a similar article that focused more on sociocultural explanations for gender differences (Brescoll and LaFrance, 2004).

The image of women is not only influenced by what is said or not said, but also by how it is said. In grammatical gender languages, such as German, French, or Russian, nouns and pronouns have masculine and feminine forms and thus differentiate for gender, for instance, “he” vs. “she” or “hero” vs. “heroine.” However, when referring to mixed-gender groups, to persons with unknown gender or persons whose gender is irrelevant, “masculine generics” are used, i.e., grammatically masculine nouns and pronouns (Hellinger and Bussmann, 2003). In contrast, gender-inclusive language makes explicit reference to women and men (word pairs, e.g., “he or she,” “firemen and firewomen”) or uses gender-neutral forms (e.g., “they,” “firefighters,” Stahlberg et al., 2007).

Past research has revealed that gender-inclusive language makes women more visible than masculine generics do (Stahlberg et al., 2007). When gender-inclusive forms are used, people assume percentages of women in a profession to be higher than when masculine generics are used (Braun et al., 2005) and more female exemplars of a category are named (Stahlberg et al., 2001; Merkel et al., 2012) or drawn (Bojarska, 2011). Most research of this kind investigated common social roles (e.g., student, professor, physician, Bojarska, 2011) and only some studies tested more prominent roles (e.g., musician, politician, Stahlberg et al., 2001). In one investigation, German participants listed more women when asked to name their favorite “heroine or hero in a novel”^1^ (word pair form; German: *Romanheldin oder Romanheld*) than those who were asked to name their favorite “hero in a novel” (*Romanheld*; Stahlberg and Sczesny, 2001). In addition to the impact of language on mental representations of women and men, recent research has shown that after reading a text with gender-inclusive wording, speakers used more gender-inclusive forms themselves in a fill-in-the-blank task (Koeser et al., 2015).

While most research focused on the effects of gender-inclusive language on perceivers (readers or listeners), there is much less research on the effects of this usage on producing such language in writing or speaking. One study that addressed effects of gender-inclusive language on users showed that participants who were made to employ forms such as *he or she* in a sentence-completion task imagined more female characters as protagonists of the sentences they were completing than participants who were made to use generic *he* (Hamilton, 1988). The author does not explain the mechanisms underlying this effect, but it is conceivable that speakers’ own use of gender-inclusive forms enhances the cognitive availability (Tversky and Kahneman, 1973) and causes a more intensive processing of these forms and of their referents.

We were interested in the impact of gender-inclusive language in media texts on mental representations of women and men and in the mechanism underlying this impact. To this end, we investigated reactions to newsworthy, exceptional social roles that are often dealt with in the media: hero and murderer. Both social roles attract much attention and have similarly low percentages of women (ca. 10–20%). In the US, only 9% of the recipients of the Carnegie Hero Medal for saving others are women, and in Germany only about 20% of similar medals are awarded to women. This may be because there are fewer women in professions such as firefighters, soldiers, or police officers—jobs involving dangerous situations where jobholders can act heroically. As for murderers, in 2014 women committed 11% of all homicides in the US (Federal Bureau of Investigation, 2015); in Germany, where the present study was conducted, it was 9% (Statistisches Bundesamt, 2015).

As mentioned above, reading a gender-inclusive text provoked more use of such language in a fill-in-the-blank task (Koeser et al., 2015). To replicate and extend this finding, we examined whether the effect on language use also held for more natural forms of language production, namely for texts written entirely by the participants themselves. We presented participants with “a short science-based press release,” and asked them to summarize the text in their own words and to indicate the percentage of women in the described social role. We expected participants summarizing a gender-inclusive text to use more word pairs and other gender-fair forms than those summarizing a text with masculine generics (Hypothesis 1). In line with past research (Stahlberg et al., 2001), we expected that reading a text with gender-inclusive forms would result in higher estimates of the percentage of women in the respective roles than reading a text with masculine generics (Hypothesis 2).

In addition, and more importantly, we examined whether participants’ own use of gender-inclusive language would lead to a higher perceived percentage of women in a given social role. In the present study, we aimed at eliciting gender-inclusive forms by having participants read a text containing such forms and by asking them to summarize its content in their own words. Thus, we directly manipulated only the text, but not participants’ language use *per se*. We expected that receiving a message in gender-inclusive wording would enhance speakers’ inclination to use such forms themselves (Koeser et al., 2015). This use, in turn, was expected to result in higher estimates of the percentage of women in the respective roles than reading a text with masculine generics (Hamilton, 1988). In other words, we expected speakers’ own use of gender-inclusive forms to mediate the relationship between the forms appearing in the text and mental representations of women in the role described in the text (Hypothesis 3).

The present investigation integrates hitherto separate lines of research on gender-inclusive language by studying three effects in the framework of one experiment: (1) the effect of reading gender-inclusive forms on speakers’ own language use, (2) the effect of reading gender-inclusive forms on mental representations of women, and (3) the effect of own language use on mental representations of women. By integrating these aspects in one experiment, the present study enables identifying the process underlying effects of gender-inclusive language. In this way, it contributes to a more comprehensive understanding of how gender-inclusive language leads to a reduction of gender stereotyping and discrimination (Sczesny et al., 2016).

## Materials and Methods

### Participants and Design

We recruited participants for an online study via a local newspaper, university mailing lists, and during the *Long Night of Scientists* in Jena, Germany (an event popularizing science among the general public). Participants were 267 native speakers of German. After deleting answers of one participant who took 17 h, the average time for completing the survey was 20 min (*SD* = 17 min). We deleted six participants who completed the survey in less than 3 min (-1 *SD*) and five who took more than 54 min (+2 *SD*). The final sample consisted of 256 persons (170 women, 86 men). Participants were of different ages (range: 14–82 years, *M* = 32.30, *SD* = 14.32) and most of them (68%) were living in the German state of Thuringia. About half (55%) of them were university students from different departments.

The experiment had a 2 (social role: heroes vs. murderers) × 2 (linguistic form: masculine generics vs. word pairs) design, with use of gender-inclusive language and estimated percentage of female heroes/murderers as dependent variables. The use of gender-inclusive language in participants’ own writing served as a potential mediator of the relationship between linguistic forms used in the text and the estimated percentage of female heroes/murderers.

### Procedure and Measures

The study was carried out in accordance with the recommendations of Swiss and German Human Research Acts. It was presented as “a study on the perception of science-based press releases.” After providing their informed consent and indicating their age and gender, participants read one (randomly chosen) of four versions of a 250-word media text about the socialization of (1) heroes, (2) heroines and heroes, (3) murderers, or (4) murderesses and murderers. The texts were identical except for the role nouns, which varied in the heading and in three other places in the text. Each text was about a study that attempted to identify factors explaining why some people become heroes/murderers (see Supplementary Materials). After reading the text, participants were asked to sum it up in their own words in 3–4 sentences.

To rule out that the text quality differed depending on linguistic forms used, we asked participants whether the information in the text was credible (accurate, credible, reliable, reflecting reality, α = 0.88) and whether the text was informative (convincing, informative, should be published, relevant, α = 0.87; scale: 1 = *totally disagree*, 7 = *fully agree*). The respective analyses showed that the texts were perceived as similarly credible and informative in both linguistic versions (*F*s < 2.60, *p*s > 0.10, $\eta_{p}^{2}$ < 0.01). The texts about heroism were perceived as more credible than the texts about murderers, *F*(1,251) = 8.23, *p* = 0.004, $\eta_{p}^{2}$ = 0.03, but the texts about murders were perceived as more informative, *F*(1,251) = 8.23, *p* = 0.004, $\eta_{p}^{2}$ = 0.03. No interactions were observed, *F*s < 1.

Then we asked five “memory questions,” including the dependent variable. The questions concerned facts and numbers that appeared in the text (How many participants took part in the study? What hobby did most of them have? How well did most of them do at school?), but also “What proportion of heroic deeds/murders are committed by women?”

Finally, participants answered some additional questions, specified demographic data^2^, were debriefed, and given the possibility to make comments. Those who left their e-mail addresses had the chance to win three prizes of €20 each.

## Results

### Use of Gender-Inclusive Language

After reading about heroism or murders, participants were asked to summarize the text in their own words. We coded the linguistic forms they used to refer to the roles of hero and murderer. The three linguistic forms most often used to describe the protagonists of the texts were: masculine forms (singular or plural, e.g., German *Helden* “heroes”), word pairs (e.g., German *Heldinnen und Helden* “heroines and heroes”), and neutralization (e.g., German *Menschen* “humans,” *Leute* “people,” *Personen* “persons”). For each participant we built an index: we computed the proportion of gender-inclusive language by dividing all gender-inclusive forms (word pairs and neutralization) by all forms used for person reference (both gender-inclusive and masculine). The resulting index ranged from 0 to 1; the higher the index, the more gender-inclusive forms were used by the participant.

**FIGURE 1:**
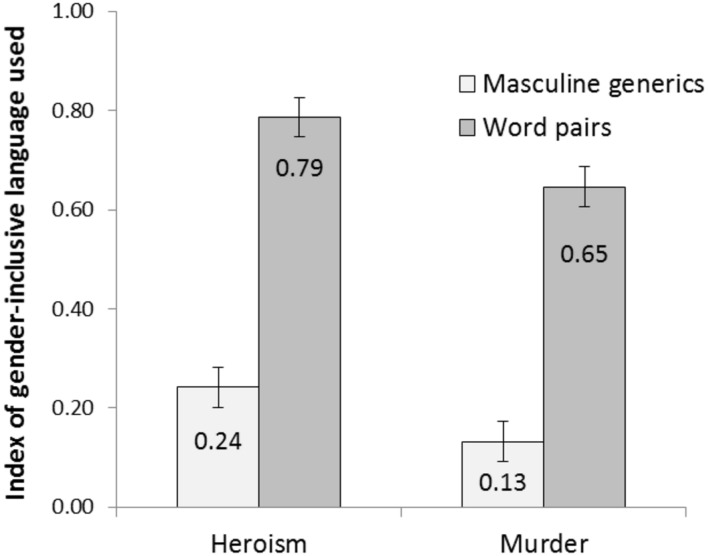
**Index of gender-inclusive language (word pairs, neutralizing words) used in the summaries by text topic (heroism vs. murder) and linguistic forms in the text (masculine generics vs. word pairs).** Error bars represent standard errors of the mean.

A 2 (social role: heroes vs. murderers) × 2 (linguistic form: masculine generics vs. word pairs) ANOVA showed that, in accord with Hypothesis 1, participants used more gender-inclusive language after reading texts with word pairs (*M* = 0.72, *SD* = 0.38) than after reading texts with masculine generics (*M* = 0.18, *SD* = 0.26), *F*(1,259) = 177.04, *p* < 0.001, $\eta_{p}^{2}$ = 0.42 (see **Figure 1**). Furthermore, participants used more gender-inclusive language when writing about heroism (*M* = 0.51, *SD* = 0.42) than about murders (*M* = 0.39, *SD* = 0.42), *F*(1,259) = 9.53, *p* = 0.002, $\eta_{p}^{2}$ = 0.04. The interaction of social role and linguistic form was not significant, *F* < 1.

Given the large age range of the sample (14–82 years), and the possibility of participant gender playing a role, we examined whether age and gender modulated the results. An ANCOVA showed no significant influence of gender (*F* < 1) and a small effect of age, *F*(1,246) = 3.30, *p* = 0.07, $\eta_{p}^{2}$ = 0.01: younger participants used slightly more gender-inclusive forms. The remaining results were virtually the same with or without both covariates.

### Estimated Percentage of Female Heroes and Murderers

**FIGURE 2:**
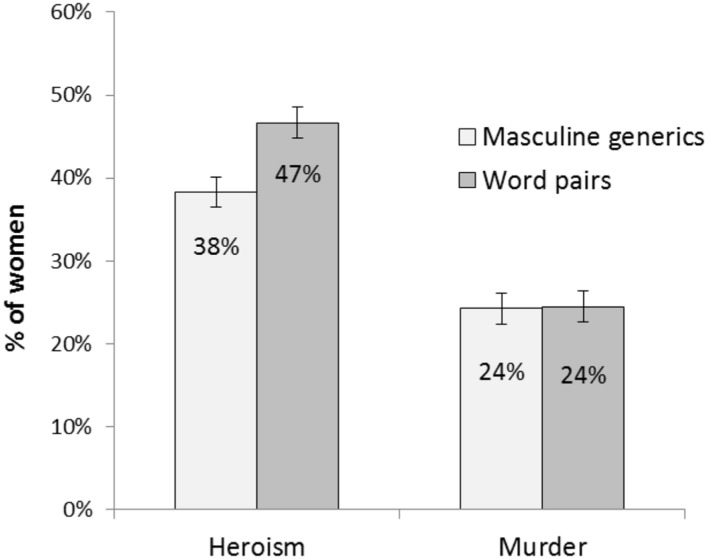
**Means of estimated percentages of female heroes and murderers by linguistic forms in the text (masculine generics vs. word pairs).** Error bars represent standard errors of the mean.

A 2 (social role: heroes vs. murderers) × 2 (linguistic form: masculine generics vs. word pairs) ANOVA revealed that, in accord with Hypothesis 2, participants who read the texts with word pairs estimated a higher percentage of women in both roles (*M* = 35.93; *SD* = 16.98) than participants who read the texts with masculine generics (*M* = 31.72; *SD* = 16.49), *F*(1,231) = 5.53, *p* = 0.02, $\eta_{p}^{2}$ = 0.02 (see **Figure 2**). Furthermore, participants estimated a higher percentage of women among heroes (*M* = 42.48; *SD* = 14.57) than among murderers (*M* = 24.37; *SD* = 13.78), *F*(1,231) = 98.87, *p* < 0.001, $\eta_{p}^{2}$ = 0.30. An interaction effect, *F*(1,231) = 5.00, *p* = 0.03, $\eta_{p}^{2}$ = 0.02, showed that those who read about “heroines and heroes” estimated a higher percentage of female heroes than those who read about “heroes,” *p* = 0.001. There was no difference in the estimated percentage of female murderers between the two language conditions, *p* = 0.94. Also, the more gender-inclusive forms participants used in their earlier summaries, the more women they perceived in the respective social role, *r*(231) = 0.26, *p* < 0.001.

An ANCOVA including participants’ gender and age again failed to show a significant influence of gender, *F*(1,246) = 2.67, *p* = 0.10, $\eta_{p}^{2}$ = 0.01, but revealed an influence of age, *F*(1,246) = 12.88, *p* < 0.001, $\eta_{p}^{2}$ = 0.05: younger participants perceived more women in the social roles described in the texts. However, the age effect was not strong and the results were the same with or without covariates.

### The Mediating Role of Gender-Inclusive Language Use

Our hypothesis predicted that gender-inclusive language would make participants use more of such language themselves, think in more gender-inclusive ways, and, in turn, estimate a higher percentage of women in the respective roles (Hypothesis 3). Therefore, we examined the potential mediating effect of own language use in the relationship between linguistic form used in the media text and perceived percentage of women in a role. As the results above showed, gender-inclusive language had a similar effect on speakers’ own use of such forms for both social roles (heroes and murderers), but affected the perceived percentage of women only among heroes. In view of this finding, we conducted a moderated mediation model (Hayes, 2013, p. 369), with social role as a moderator.

**FIGURE 3:**
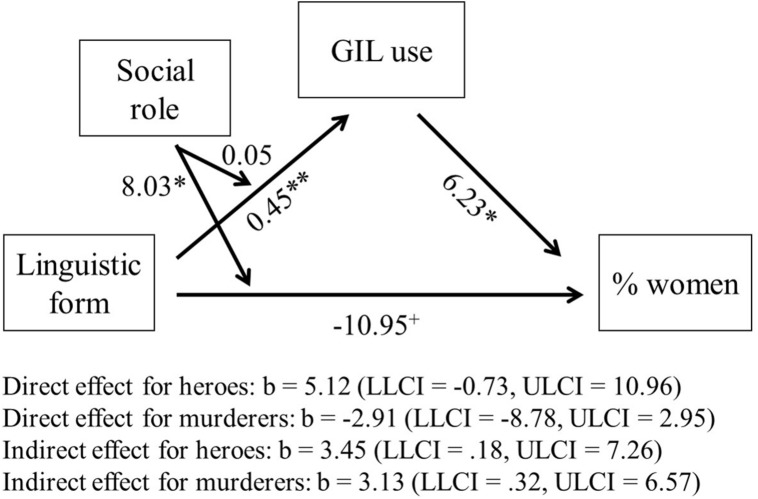
**Effect of linguistic forms in the text on estimated percentage of women in a role, mediated by the amount of gender-inclusive language (GIL) used by participants in the summaries of the texts.** *N* = 232. Unstandardized regression coefficients are shown. Linguistic form was coded as 0 = masculine generics, 1 = word pairs. Social role was coded as 0 = murderer, 1 = hero. ^+^*p* < 0.10; ^∗^*p* < 0.05; ^∗∗^*p* < 0.01.

The analysis showed that after reading the media text containing word pairs participants used more gender-inclusive forms themselves (see **Figure 3**), in line with our previous findings. The more gender-inclusive language they used, the more women they perceived in this role. As shown above, the social role described in the text did not affect participants’ own use of gender-inclusive language (no interaction of linguistic form and social role), but affected the perceived percentage of women (higher percentage of women for heroes, but not for murderers). Beyond previous findings and in accord with Hypothesis 3, the analysis revealed that when own use of gender-inclusive language was included, the direct effect of linguistic forms in the text disappeared for the category heroes and remained insignificant for murderers. Indirect (i.e., mediational) effects occurred for both roles. Thus, having read a text with word pairs, participants used more such forms themselves, and this, in turn, promoted more gender-balanced representations in both roles.

### Post-test

The main effects for estimated percentage of women in the two social roles resembled the results obtained for own language use: participants used more gender-inclusive language and estimated a higher percentage of women after reading the texts with word pairs and after reading the texts about heroism. However, gender-inclusive language increased only the estimated percentage of female heroes, but not the percentage of female murderers. It has been suggested that the number of female exemplars available in a person category may play a role for the changes in gender perceptions that are evoked by gender-inclusive language (Braun et al., 2005). Thus, the observed asymmetry in our results may be due to a different availability of female exemplars among heroes and murderers. To address this possibility, we conducted a post-test.

The post-test assessed how often people thought of women when asked about heroism and murders, without variation in language forms. We asked passers-by (*N* = 35, 21 women, 14 men, *M*_age_ = 35.17, *SD* = 14.82, age range: 19–74) in Jena, Germany, to fill out a short questionnaire in exchange for a chocolate bar. The instruction given in a booklet asked participants to name “people who behaved heroically” and, on the next page, “people who murdered someone” (order counterbalanced). Next, we explicitly asked participants to write down “women who behaved heroically” and “women who killed someone.”

The results showed that participants spontaneously named a total of 82 (*M* = 2.34) male and 12 (*M* = 0.34) female murderers, as well as 45 (*M* = 1.29) male and 22 (*M* = 0.63) female heroes. Thus, they mentioned more men than women for both roles, but the proportion of women was higher for heroes (33%) than for murderers (13%). Furthermore, participants named more female heroes than female murderers, while the opposite was true for male heroes and murderers. When asked explicitly about women, they again named more female heroes (37) than female murderers (23). In all, the post-test revealed that more exemplars of female heroes were available to the participants than of female murderers.

## Discussion

The present study shows that the language used in the media to describe social roles affects readers’ own language use and that this, in turn, can influence the social perception of groups. In line with and extending earlier research, which relied on fill-in-the-gap sentences (Koeser et al., 2015), participants used more gender-inclusive forms after reading a text with word pairs when summarizing the text in their own words. Moreover, participants who read about “heroines and heroes” estimated a higher percentage of female heroes than those who read about “heroes.” Most importantly, we found that gender-inclusive language triggered a more gender-balanced use of language, and this use resulted in a more gender-balanced perception of social roles. In this process, participants’ own language use functioned as a mediator between the language of the media texts and the mental representation of social roles. Thus, our results not only replicate and link previous findings (Hamilton, 1988; Stahlberg et al., 2001; Koeser et al., 2015), but also illuminate the mechanism behind the effects of gender-inclusive language. Participants’ own use of gender-inclusive language made them think in more gender-inclusive ways.

While readers of a text about “heroines and heroes” perceived more female heroes than readers of a text about “heroes,” the estimated percentage of women committing murder did not differ between readers of a text about “murderesses and murderers” and about “murderers.” Our post-test showed that people were aware of more female heroes than murderers. It seems that when people know some women in a given role (Becker and Eagly, 2004; Rankin and Eagly, 2008), gender-inclusive language can trigger the female exemplars that are known and can make the mental representation of a role more gender-balanced (Braun et al., 2005). In other words, language can impact speakers’ perceptions to some extent, but only within the boundaries of social reality. Other explanations for the observed effect are also conceivable. It is possible, for instance, that norm congruence plays a role here. Speaking of women as murderers might be too negative and less reconcilable with the female stereotype of a caring, nurturing, and selfless mother, than speaking of women as heroes. Perhaps gender-inclusive language makes women more visible in positive roles, but not in negative ones. However, our participants generally overrated the percentage of murderesses, which suggests that they were not trying to protect women’s image. Furthermore, earlier studies have shown that gender-inclusive language has an effect also when talking about disliked personalities (e.g., the least liked politician, Gabriel and Mellenberger, 2004; Gabriel, 2008). Still another explanation of the observed effect could be the different flexibility of the definition of both roles. Perhaps category boundaries are clearer and more straightforward for “murderers” than for “heroes.” It might be easier to extend the definition of a hero to include more women than to extend the definition of a murderer (Becker and Eagly, 2004; Franco et al., 2011).

Although the effect of gender-inclusive language on the estimated percentage of women was significant only for heroes but not for murderers, the mediation effect occurred for both roles. The mediation findings suggest that triggering people to use gender-inclusive forms in their writing can make them process information more intensively and enhance cognitive availability of these forms and their referents (Tversky and Kahneman, 1973). In addition, participants may have inferred that because they used gender-inclusive forms in their summaries themselves, there must be a considerable percentage of women in these roles. This self-perception mechanism (Fazio et al., 1977) could be operating in parallel to a more intensive processing due to own use of gender-inclusive language.

Future research should aim at replicating the obtained results and at further contributing to an explanation of the mechanisms underlying them. It could be also studied whether similar effects are present while listening to gender-inclusive language and while using it in one’s own speech. Potential mediating variables could be assessed and/or manipulated. The accessibility of exemplars, for instance, could be manipulated by carefully choosing roles that differ in this respect, but not in valence, or by experimentally making some exemplars more accessible. The influence of the roles’ valence could be studied by investigating roles with comparable numbers of known female exemplars, but with a different valence. To generalize the effects of valence, it would be advisable to include several positive and negative roles. Future research could also test whether the effect of texts in gender-inclusive language on the own use of such language and on the mental representations “spills over” to other social roles than the ones provided.

Gender-inclusive language can be used to reduce gender-stereotypic images of certain male-typed social roles in a community with a grammatical gender language (Horvath and Sczesny, 2016). Our results show that both perceiving and producing gender-inclusive language can evoke more gender-balanced mental representations of social roles. Such language can be effective not only when read or heard, but it can expand and resonate later when reproduced. The present study also suggests that reality will not be distorted if the media use more gender-inclusive language, but that this type of language may help to present women and men more equally in various social roles.

## Author Contributions

All authors listed, have made substantial, direct and intellectual contribution to the work, and approved it for publication.

## Conflict of Interest Statement

The authors declare that the research was conducted in the absence of any commercial or financial relationships that could be construed as a potential conflict of interest. The reviewer SM and handling Editor declared their shared affiliation, and the handling Editor states that the process nevertheless met the standards of a fair and objective review.

## Footnotes

1 We use the expressions “heroines” along with “heroes” as well as “murderesses” along with “murderers” to render the German wording. However, we are aware that in English these expressions are not as gender-fair as in German.

2 We also included a few measures that are not the focus of the current article. The description and basic results on these measures are available in the Supplementary Materials.
