# Supplementary material for: The Social Perception of Heroes and Murderers: Effects of Gender-Inclusive Language in Media Reports
Source: Front Psychol. 2016 Mar 22;7:369. doi: 10.3389/fpsyg.2016.00369 (PMC4801896; doi:10.3389/fpsyg.2016.00369)
Supplement: Supplementary file 1 [file Appendix.PDF]

Appendix

Translation of the stimulus text

On the socialization of **heroes**

[Other versions: **heroines and heroes/murderers/murderesses and murderers**; emphasis added]

Very few people become **heroes** in the course of their lives. As a rule, this happens suddenly and seems to be unforeseeable. Their behavior changes their own lives and those of others to an inconceivable extent. How can their deeds be explained? A scientific study investigated their socialization. It aimed at identifying factors that may explain an act of this kind. To this end, interviews were conducted with 127 **heroes** who were asked about their personal development.

The survey shows that most of them had a fairly normal childhood. The families of the interviewees were predominantly middle class. According to the analysis of the interview data, most of the interviewees had good relationships with their grandparents and other family members. At nursery school and at school they had [female and male] friends. Their academic performance at school was mostly in the average range. During adolescence, most of them had a hobby. Their hobbies varied widely, with many of the interviewees enjoying sports. As adults, they worked in different occupations. Many of them lived in a partnership with children and led an ordinary family life.

In summary, it was impossible to identify definite factors in the socialization of **heroes**, which could have predicted their exceptional behavior in adult age. However, the study showed that an explanation could often be found in the specific situation. Most of the interviewees stated that, while being in the situation, they did not think what their act meant or the consequences it might have had, they were guided by their emotions.

Other measures

After reading the text participants were asked, additionally to evaluating its credibility, whether the text threatened the image of women, and whether the text threatened the image of men. The results showed no significant effects on this measure and a general floor effect (M_{threat_women} = 1.35, M_{threat_men} = 1.43). For exploratory reasons we included the following question among the "memory questions": "Do you think that there were more or less murders/heroic acts in 2012 than in the previous year?" There were no significant differences on this measure. Subsequently, participants answered an open question "Who did you imagine while reading the text?" There were no significant differences between conditions. A descriptive result was that participants reading about "heroines and heroes" imagined slightly fewer cases of rescuing others (e.g., from a fire or drowning) and more cases of civic courage and everyday help than participants reading about "heroes". For the texts about murders, there were no differences between texts in the two linguistic forms and many participants wrote that they did not imagine any specific person or event.

Finally, after sampling all dependent variables, we explored several potential moderators: centrality of own gender, modern sexism, attitudes towards gender-fair language, motivation to respond in an unprejudiced way, and political attitudes. Unfortunately, these variables could not be treated as moderators, as there were effects of the manipulation on them. Future studies should measure such moderators (weeks) before sampling the dependent variables. For exploratory reasons, we computed ANCOVAs including these measures. The analyses yielded virtually the same results as the ones described in ANOVAs in the Results section.
